# Contrast-Enhanced Spectral Mammography-Based Radiomics Nomogram for Identifying Benign and Malignant Breast Lesions of Sub-1 cm

**DOI:** 10.3389/fonc.2020.573630

**Published:** 2020-10-30

**Authors:** Fan Lin, Zhongyi Wang, Kun Zhang, Ping Yang, Heng Ma, Yinghong Shi, Meijie Liu, Qinglin Wang, Jingjing Cui, Ning Mao, Haizhu Xie

**Affiliations:** ^1^ School of Medical Imaging, Binzhou Medical University, Yantai, China; ^2^ Department of Radiology, Yantai Yuhuangding Hospital, Affiliated Hospital of Qingdao University, Yantai, China; ^3^ Department of Breast Surgery, Yantai Yuhuangding Hospital, Affiliated Hospital of Qingdao University, Yantai, China; ^4^ Department of Pathology, Yantai Yuhuangding Hospital, Affiliated Hospital of Qingdao University, Yantai, China; ^5^ Collaboration Department, Huiying Medical Technology, Beijing, China

**Keywords:** nomogram, small lesion, breast, radiomics, contrast-enhanced spectral mammography

## Abstract

**Objectives:**

To develop a radiomics nomogram that incorporates contrast-enhanced spectral mammography (CESM)-based radiomics features and clinico-radiological variables for identifying benign and malignant breast lesions of sub-1 cm.

**Methods:**

This retrospective study included 139 patients with the diameter of sub-1 cm on cranial caudal (CC) position of recombined images. Radiomics features were extracted from low-energy and recombined images on CC position. The variance threshold, analysis of variance (ANOVA) and least absolute shrinkage and selection operator (LASSO) algorithms were used to select optimal predictive features. Radiomics signature (Rad-score) was calculated by a linear combination of selected features. The independent predictive factors were identified by ANOVA and multivariate logistic regression. A radiomics nomogram was developed to predict the malignant probability of lesions. The performance and clinical utility of the nomogram was evaluated by receiver operating characteristic (ROC) curve, calibration curve, and decision curve analysis (DCA).

**Results:**

Nineteen radiomics features were selected to calculate Rad-score. Breast imaging reporting and data system (BI-RADS) category and age were identified as predictive factors. The radiomics nomogram combined with Rad-score, BI-RADS category, and age showed better performance (area under curves [AUC]: 0.940, 95% confidence interval [CI]: 0.804–0.992) than Rad-score (AUC: 0.868, 95% CI: 0.711–0.958) and clinico-radiological model (AUC: 0.864, 95% CI: 0.706–0.956) in the validation cohort. The calibration curve and DCA showed that the radiomics nomogram had good consistency and clinical utility.

**Conclusions:**

The radiomics nomogram incorporated with CESM-based radiomics features, BI-RADS category and age could identify benign and malignant breast lesions of sub-1 cm.

## Introduction

Breast cancer is a malignant tumor that endangers women’s health and quality of life. The development of medical imaging technology and the widespread use of breast cancer screening have gradually increased the detection rate of small breast lesions (1). For small lesions, malignant signs are not obvious due to the lack of specificity in imaging features. Existing imaging methods have difficulty making accurate qualitative diagnosis; thus, breast lesions recognized as breast imaging reporting and data system (BI-RADS) category 4 or 5 are usually recommended for biopsy (2). However, the results of biopsy are affected by the biopsy site and material (3), and the small amount of biopsy tissue cannot cover the entire lesion, preventing biopsy from fully reflecting the heterogeneity of the whole lesion. Moreover, the small size of lesions brings difficulty for clinicians in performing a successful biopsy, and as an invasive examination, biopsy has the risk of causing serious complications, such as severe bleeding and infection (4, 5). Therefore, using non-invasive methods to discriminate the nature of small lesions and help radiologists and clinicians make accurate diagnosis and clinical decision is important.

Mammography is a common examination method for breast diseases, but has difficulty finding small lesions, especially in dense breasts. Initial results showed that contrast-enhanced spectral mammography (CESM) had higher specificity in the diagnosis of breast cancer than mammography (6). Breast ultrasound can evaluate breast lesions with the combination of morphology and blood flow, but is insensitive to calcification. Breast magnetic resonance imaging (MRI) has high sensitivity (90.1%) and accuracy (82.8%) in distinguishing breast lesions (7), but showed lower accuracy on small lesions than on advanced lesions (8). Moreover, MRI has not been used as a routine examination method due to its long imaging time and contraindications.

CESM is an emerging technology that combines intravenous iodine contrast enhancement with digital mammography. After intravenous contrast injection, high-energy and low-energy mammography are taken. Recombined images are generated by subtracting the unenhanced tissue on post-processing system. The low-energy images are equivalent to mammography, showing various signs of lesions such as calcification and distorted structure. The recombined images retain the abnormal enhanced area, and the degree of lesion enhancement indirectly reflects the blood supply of the lesion. Related research showed that CESM had similar sensitivity (94% vs. 99%) and higher positive predictive value (93% vs. 60%) in detecting breast cancer compared with MRI (9).

In clinical settings, using above-mentioned methods to determine the nature of small breast lesions is still a challenge (10), and the accuracy depends on the experience of radiologists. In 2012, the concept of radiomics was first proposed by Lambin et al. (11). A goal of radiomics is to convert medical images into collectable, high-fidelity, and high-throughput data, and use radiomics features to develop predictive models and support clinical decisions (12, 13). To some extent, radiomics has solved the problem of quantitative assessment of tumor heterogeneity and has shown great advantages in clinical application, such as lesions discrimination, prediction of cancer molecular subtypes, and prediction of lymph node metastasis (14–18).

At present, studies that focus on classification of small breast lesions are very limited. Our research extracted radiomics features from CESM images and aimed to establish a radiomics nomogram based on radiomics signature and clinico-radiological predictive factors to automatically identify benign and malignant breast lesions of sub-1 cm.

## Materials and Methods

### Patients and Cohorts

**Figure 1:**
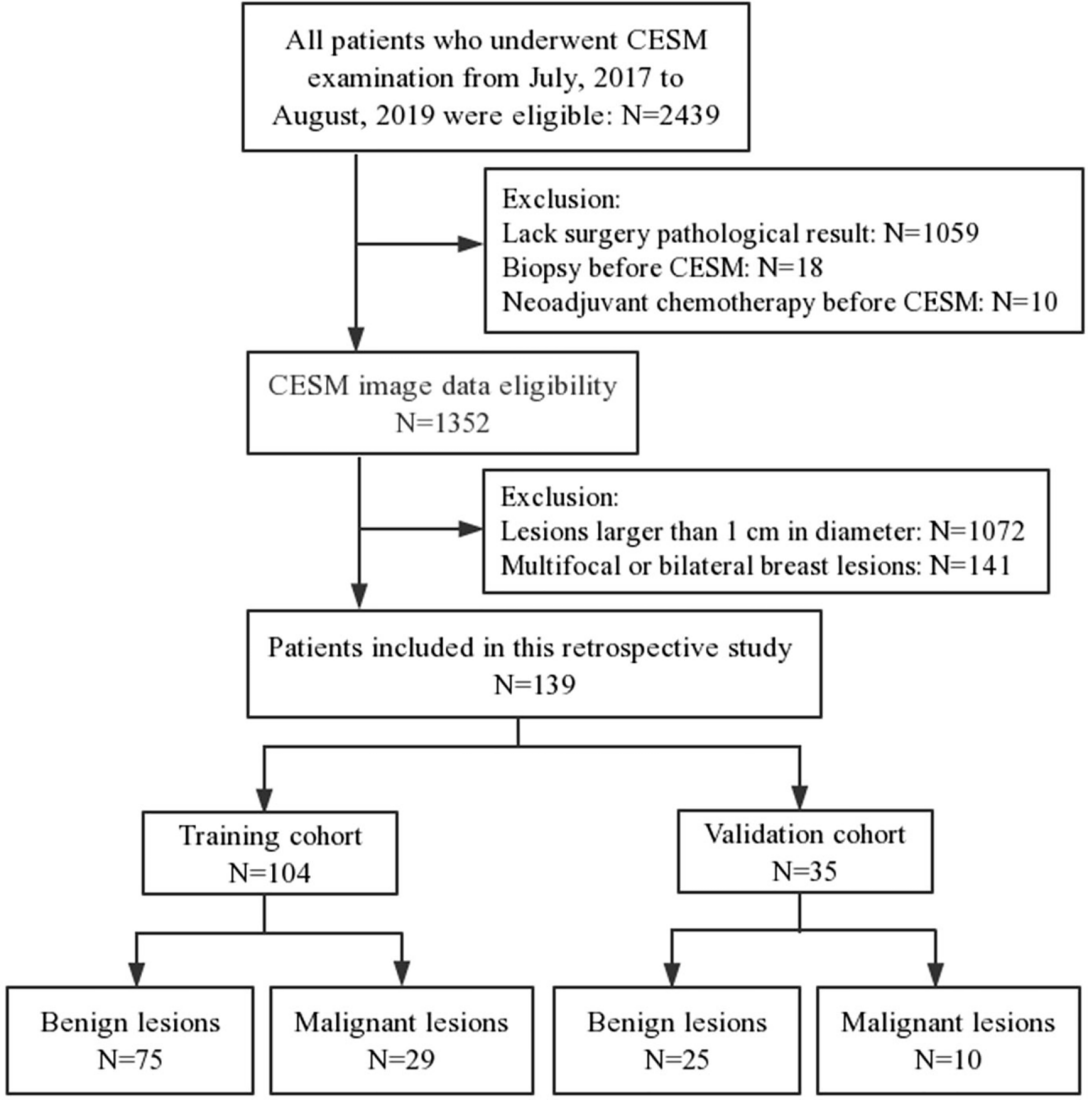
Flow chart of patients enrolment.

This retrospective study was approved by the institutional Ethics Committee. A total of 2,439 patients underwent CESM examination from July 2017 to August 2019. The inclusion criteria included (a) the diameter of lesion was less than 1 cm on cranial caudal (CC) position of recombined image, (b) diagnosed with a definite pathology result, and (c) surgery within 14 days after CESM examination. The exclusion criteria included (a) multifocal or bilateral breast lesions, (b) biopsy before CESM examination, and (c) patients underwent neoadjuvant chemotherapy before CESM examination. Finally, 139 women (mean age=46.20 ± 11.02 years; range=17–71 years) were enrolled in this study, including 39 malignant lesions and 100 benign lesions (**Figure 1**). The patients were separated into a training cohort with 104 lesions (75 benign and 29 malignant lesions) and a validation cohort with 35 lesions (25 benign and 10 malignant lesions) randomly with the ratio of 8:2.

### CESM Image Acquisition

All patients underwent CESM examination on a full-digital breast machine (Senographe DS Senobright, GE Healthcare). The contrast agent Omnipaque 300 (GE Healthcare, Inc., Princeton, NJ) was injected into the upper arm vein with the dose of 1.5 ml/kg and the injection flow rate of 3.0 ml/s. The mammogram was obtained after injecting the contrast agent for 2 min. Same as the compression method of mammography, the CC position and mediolateral oblique (MLO) position photography were performed on bilateral breast. After low-energy exposure and high-energy exposure, eight images were collected within 5 min, including four low-energy and four high-energy images. Then, four recombined images were acquired by post-processing system.

### Clinico-Radiological Characteristics

The diameters of lesions were measured on CC position of recombined images. The CESM images were evaluated by two radiologists (reader 1 with 10 years of experience on breast imaging, and reader 2 with 6 years of experience on breast imaging) according to BI-RADS. Differences in BI-RADS category between the two readers were determined by another radiologist with 15 years of experience on breast imaging. Considering that the background parenchymal enhancement (BPE) and breast density may be the risk factors of breast cancer (19, 20), BPE was evaluated on MLO position of recombined images in bilateral breast according to enhancement range (21) and breast density was evaluated on MLO position of low-energy images in bilateral breast according to the amount of fibroglandular tissue.

### Image Segmentation and Radiomics Feature Extraction

**Figure 2:**
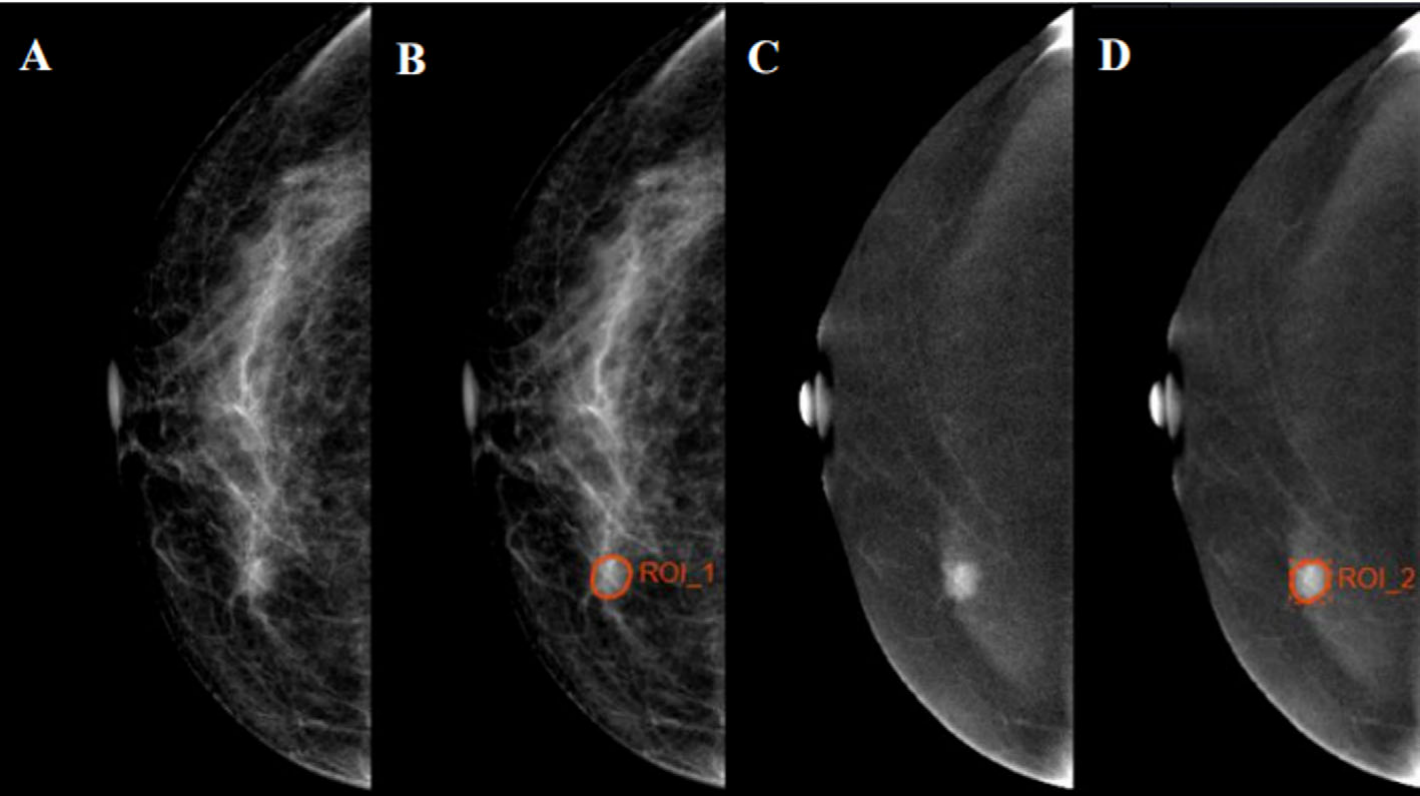
Examples of regions of interest (ROIs) segmentation on contrast-enhanced spectral mammography (CESM) images. **(A, C)** The low-energy and recombined images on cranial caudal (CC) position, respectively. **(B, D)** The ROIs of breast lesions were drawn manually on low-energy and recombined images, respectively.

All Digital Imaging and Communications in Medicine (DICOM) images were acquired from the Picture Archiving and Communication System (PACS) and uploaded to Radcloud (Huiying Medical Technology Co., Ltd.). Reader 1, who was blind to the pathology reports, identified the regions of interest (ROIs) and segmented manually on low-energy and recombined images of CC position. A sample of segmentation process is shown in **Figure 2**. Data preprocessing was conducted before features extraction by standardizing the images. Quantitative radiomics features were extracted from ROIs on Radcloud platform (http://radcloud.cn/). The extracted features were divided into three categories: first-order statistics, shape- and size-based, and texture features.

To calculate the intra- and inter-observer agreement of feature extraction, 30 breast lesions were selected randomly by statistical software. Reader 2 used the same method to extract radiomics features, and after 3 months, reader 1 repeated features extraction. Inter- and intra-correlation coefficients (ICCs) were calculated to assess the reproducibility of radiomics features, and ICCs > 0.75 was considered to represent good agreement.

### Radiomics Feature Selection and Radiomics Signature Construction

The variance threshold, analysis of variance (ANOVA), and least absolute shrinkage and selection operator (LASSO) methods were used to reduce the redundant features and select optimal radiomics features. The threshold of variance threshold method was 0.8; thus, the eigenvalues of the variance smaller than 0.8 were removed. In ANOVA method, all the features that showed significant differences (p < 0.05) between benign and malignant lesions were included. For LASSO algorithm, the optimal LASSO alpha parameter was set by five-fold cross validation and radiomics features with non-zero coefficients within the training cohort were finally selected.

The radiomics signature (Rad-score) of each lesion was calculated by a linear combination of selected features, which were weighted by their respective coefficients.

### Development of the Radiomics Nomogram

Using data from the training cohort, one-way ANOVA and multivariate logistic regression were performed to analyze independent predictive factors related to the identification of benign and malignant breast lesions, including clinico-radiological characteristics (age, tumor diameter, BI-RADS category, BPE, and density) and Rad-score. After multivariate logistic regression, variables with P<0.05 were considered as independent predictive factors. A radiomics nomogram was developed by multivariate logistic regression. Rad-score and clinico-radiological model were also developed in the training cohort to estimate the value of radiomics.

### Validation of the Nomogram

The receiver operating characteristic (ROC) curves and the area under curves (AUC) were performed to evaluate the predictive performance of the nomogram in the training and validation cohorts. The calibration curves were used to evaluate the agreement between the observed results and predicted probability. The clinical utility of the nomogram was evaluated through quantifying the net benefit under different threshold probabilities in the validation cohort by decision curve analysis (DCA). Net benefit is defined as the proportion of true positive minus the proportion of false positive as weighted by the relative risk of false positive and false negative results. The formula of net benefit is as follows:

$$\text{Net}\;\text{benefit}\;=\;\frac{\text{True}\;\text{positive}\;\text{count}}{\text{n}}-\frac{\text{False}\;\text{positive}\;\text{count}}{\text{n}}(\frac{Pt}{1-Pt}),$$

where n is the number of patients; and Pt is the threshold probabilities.

### Statistical Analysis

The training cohort (80%) was used to develop radiomics nomogram, while the validation cohort (20%) was only utilized for assessment. The pathology results were used as gold standard in classifying benign and malignant lesions. Continuous variables (age and diameter) were compared by t-test, while qualitative variables (BI-RADS category, BPE, and density) were analyzed by chi-square test or Fisher’s exact test. One-way ANOVA and multivariate logistic regression analysis were used to select the significant predictive factors in identifying benign and malignant lesions. The DeLong test was used to compare the difference between AUCs in Rad-score, clinico-radiological model, and radiomics nomogram. The statistical analysis was performed in R software (version 3.4.1) and SPSS (version 26). The “glmnet,” “glm,” “rms,” “pROC,” “CalibrationCurves,” and “DecisionCurve” packages were used. P < 0.05 was regarded as a statistically significant difference.

## Results

### Clinico-Radiological Characteristics

A total of 27.9% and 28.6% of patients were found with malignancy in the training and validation cohorts, respectively, with no significant difference (p=0.938). The clinico-radiological characteristics between benign and malignant lesions of the training and validation cohorts are shown in **Table 1**. Significant differences in age (p=0.001), diameter (p=0.011), and BI-RADS category (p<0.001) but no significant differences in BPE (p=0.393) and density (p=0.221) were found between benign and malignant lesions in the training cohort.

**Table 1:** Clinico-radiological characteristics in the training and validation cohorts.

	Training cohort (n=104)		*P*	Validation cohort (n=35)		*P*
	Benign(n=75)	Malignant(n=29)		Benign(n=25)	Malignant(n=10)	
Age, years (mean ± SD)	43.87 ± 10.63	51.90 ± 9.16	0.001*	42.64 ± 10.37	56.10 ± 7.88	0.001*
Diameter, cm (mean ± SD)	0.81 ± 0.17	0.90 ± 0.14	0.011*	0.81 ± 0.14	0.96 ± 0.05	<0.001*
BI-RADS category			<0.001*			0.039*
3	8	0		3	0	
4A	38	2		14	2	
4B	20	11		4	4	
4C	8	11		4	2	
5	1	5		0	2	
BPE			0.393			0.646
minimal	26	13		9	5	
mild	46	16		15	5	
moderate	3	0		1	0	
marked	0	0		0	0	
Density			0.221			0.157
entirely fatty	7	3		0	1	
scattered fibroglandular	56	25		22	9	
heterogeneously dense	12	1		3	0	
extremely dense	0	0		0	0	

BI-RADS, Breast Imaging Reporting and Data System; BPE, Background Parenchymal Enhancement; SD, standard deviation. *P < 0.05.

### Radiomics Feature Selection and Radiomics Score Construction

**Figure 3:**
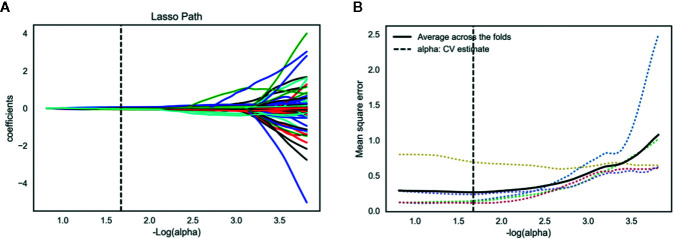
Lasso algorithm for radiomics features selection. **(A)** Least absolute shrinkage and selection operator (LASSO) coefficient profiles of the 103 features. The y-axis represents coefficient of each feature. The optimal value of alpha was 0.0214, and the optimal –log(alpha) was 1.67, where 19 features with non-zero coefficient were selected. **(B)** Mean square error path using five-fold cross-validation.

The inter- and intra-observer reproducibility of features extraction has achieved with ICC > 0.75 both between the two different radiologists and the same radiologist 1. A total of 2,056 features were selected from 2,818 radiomics features using variance threshold method. Then, 103 features were further selected by ANOVA method. Finally, the optimal 19 features were selected with non-zero coefficients in LASSO logistic regression (**Figure 3** and **Table 2**).

**Table 2:** Least absolute shrinkage and selection operator (LASSO) coefficient profiles of the 19 features.

Radiomics Features	Coefficients
Low-energy_wavelet-LLL_firstorder_Range	0.089292756
Low-energy_wavelet-HLL_firstorder_10Percentile	−0.021570008
Recombined_wavelet-LHH_firstorder_Median	0.023354281
Recombined_wavelet-HHH_glszm_GrayLevelNonUniformity	0.022899496
Low-energy_wavelet-HHH_ngtdm_Contrast	−0.025332901
Low-energy_wavelet-LHL_glszm_LowGrayLevelZoneEmphasis	−0.082688563
Recombined_wavelet-LLH_firstorder_Kurtosis	−0.024670295
Recombined_wavelet-HHH_glszm_SizeZoneNonUniformityNormalized	−0.03792366
Recombined_wavelet-LHH_glrlm_RunVariance	0.04721272
Low-energy_wavelet-LLH_firstorder_Minimum	−0.019677652
Low-energy_wavelet-LLH_ngtdm_Strength	0.03722361
Recombined_wavelet-LHL_glszm_GrayLevelNonUniformityNormalized	0.018366436
Low-energy_wavelet-LHL_gldm_HighGrayLevelEmphasis	−0.007483205
Recombined_wavelet-HLL_glszm_SmallAreaLowGrayLevelEmphasis	−0.062991217
Recombined_wavelet-HHH_glcm_SumSquares	−0.003659382
Recombined_wavelet-HHL_glszm_SmallAreaHighGrayLevelEmphasis	0.009975399
Low-energy_wavelet-HLH_gldm_SmallDependenceLowGrayLevelEmphasis	−0.013119935
Low-energy_wavelet-HHH_glrlm_ShortRunEmphasis	−0.024312171
Recombined_wavelet-HHH_gldm_GrayLevelVariance	−0.026478317

glszm, gray level size zone matrix; ngtdm, neighborhood gray tone difference matrix; glrlm, gray level run length matrix; gldm, gray level dependence matrix; glcm, gray level co-occurrence matrix.

Rad-score of each lesion was calculated by the 19 radiomics features. Rad-score showed a significant difference between benign and malignant lesions in the training cohort (p<0.001), and the optimal cutoff value was 0.376 in distinguishing benign and malignant lesions.

### Development of the Nomogram

**Figure 4:**
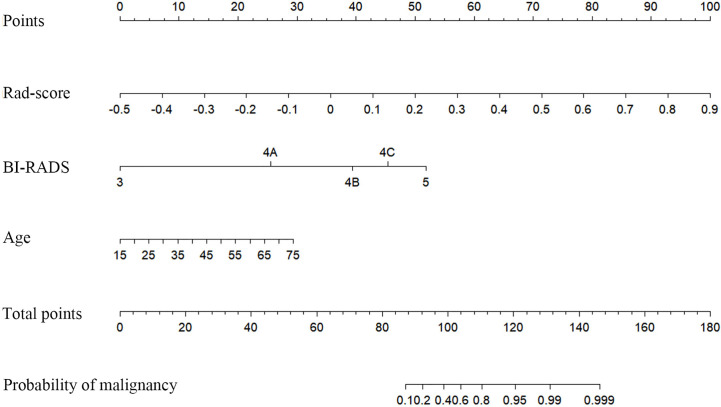
Radiomics nomogram with Rad-score, Breast imaging reporting and data system (BI-RADS) category, and age incorporated.

In the training cohort, diameter (p=0.017), BI-RADS category (p<0.001), and age (p<0.001) were input to multivariate logistic regression after one-way ANOVA. In the multivariate logistic regression, Rad-score and age (both p<0.05) were proven to be the independent predictive factors in identifying benign and malignant lesions. The radiomics nomogram was developed with Rad-score, BI-RADS category, and age (**Figure 4**). To estimate the value of radiomics nomogram, clinico-radiological model was built with BI-RADS category and age.

### Validation of the Nomogram

**Figure 5:**
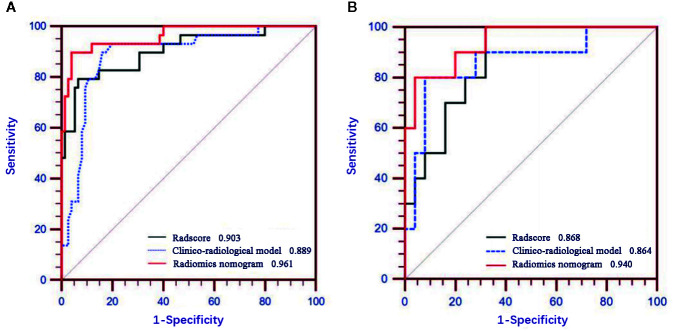
Receiver operating characteristic (ROC) curves of Rad-score, clinico-radiological model and radiomics nomogram in the training **(A)** and validation **(B)** cohorts.

**Figure 5** displays the ROCs of Rad-score, clinico-radiological model, and radiomics nomogram in the training and validation cohorts. The optimal cutoff value of Rad-score, clinico-radiological model, and radiomics nomogram was 0.376, 0.369 and 0.512, respectively. The AUCs of Rad-score, clinico-radiological model and radiomics nomogram in the training cohort were 0.903 (95% CI, 0.830–0.953), 0.889 (95% CI, 0.812–0.942) and 0.961 (95% CI, 0.904–0.989), respectively; and AUCs in the validation cohort were 0.868 (95% CI, 0.711–0.958), 0.864 (95% CI, 0.706–0.956), and 0.940 (95% CI, 0.804–0.992), respectively. DeLong test showed that there was significant difference between clinico-radiological model and radiomics nomogram (p=0.019) in the training cohort, but showed no significant difference in the validation cohort (p=0.153). The radiomics nomogram showed higher accuracy and specificity than Rad-score and clinico-radiological model in predicting benign and malignant lesions (**Table 3**).

**Table 3:** Predictive performance of three models.

	Training cohort			Validation cohort		
	AUC(95% CI)	Sensitivity	Specificity	AUC(95% CI)	Sensitivity	Specificity
Rad-score	0.903 (0.830–0.953)	0.793	0.933	0.868 (0.711–0.958)	0.700	0.800
Clinico-radiological model	0.889 (0.812–0.942)	0.897	0.840	0.864 (0.706–0.956)	0.800	0.920
Radiomics nomogram	0.961 (0.904–0.989)	0.897	0.960	0.940 (0.804–0.992)	0.800	0.960

**Figure 6:**
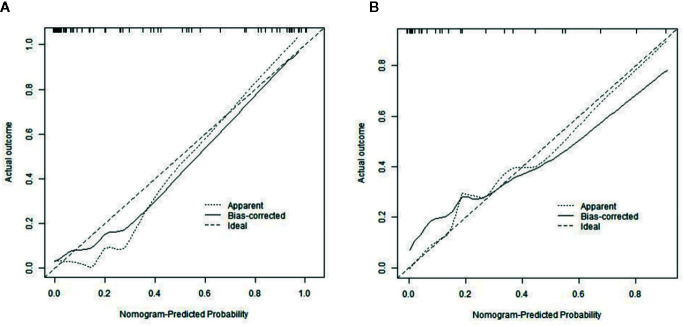
Calibration curves of radiomics nomogram in the training **(A)** and validation **(B)** cohorts. The diagonal line represents the perfect prediction of the radiomics nomogram. The black solid line represents the calibration curve of radiomics nomogram. The calibration curves are close to the diagonal line both in the training and validation cohorts, which shows that the prediction probability have good agreement with the actual probability.

**Figure 7:**
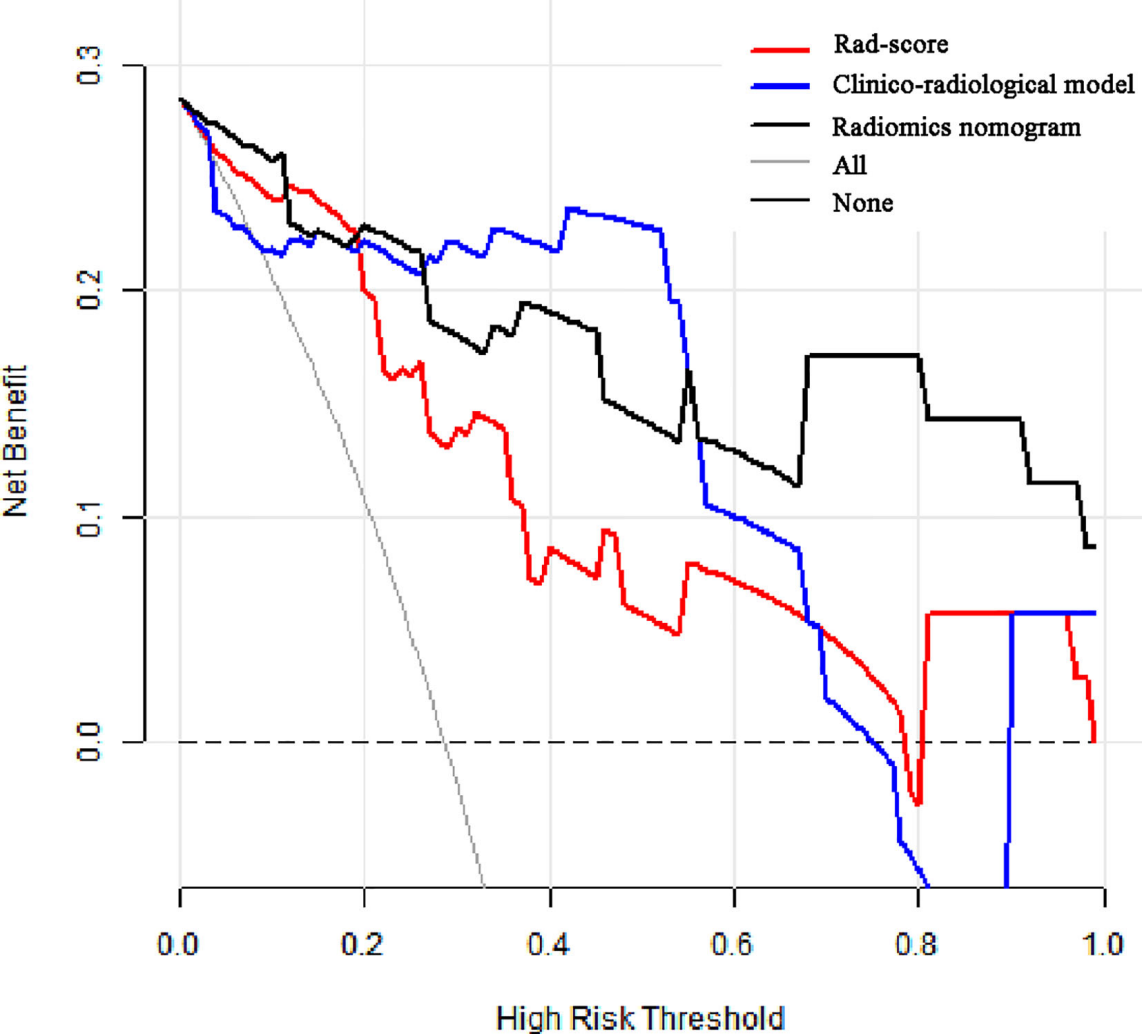
Decision curve analysis (DCA) for the prediction models in the validation cohort. The y-axis represents the net benefits, while the x-axis represents the threshold probability. The red line represents the Rad-score. The blue line represents the clinico-radiological model. The black line represents the radiomics nomogram. The gray line represents the assumption that all patients were included in benign group. The dotted black line represents the assumption that all patients were included in malignant group. The decision curve shows that radiomics nomogram can add more net benefit than “none” or “all” treatment with the threshold probability range from 0 to 1.0.

**Figure 8:**
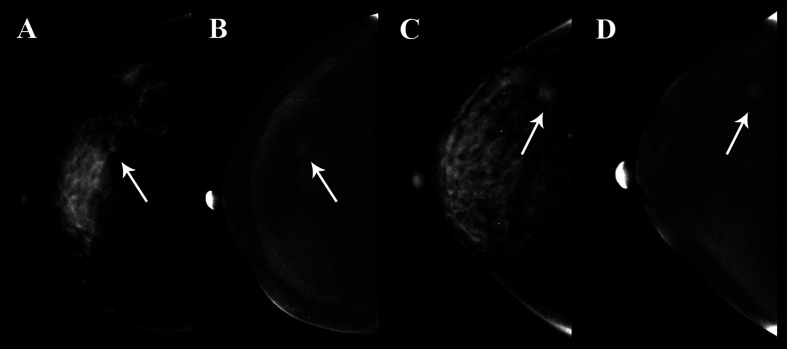
Clinical use of two patients who were both diagnosed with BI-RADS 4B category. The breast lesions of the two patients had similar imaging features on the contrast-enhanced spectral mammography (CESM) images, and the arrow points were the lesions of the two patients. **(A, B)** A 35-year-old woman, whose malignancy probability was calculated less than 10% by nomogram, was confirmed as fibroadenoma by pathological examination. **(C, D)** A 41-year-old woman, whose malignancy probability was calculated at about 62% by nomogram, was confirmed as invasive ductal carcinoma by pathological examination.

The calibration curves of radiomics nomogram demonstrated good consistency between predictive outcome and observation in the training and validation cohorts (**Figure 6**). The DCA indicated that radiomics nomogram could add more net benefits than “all treatment” or “none treatment” with the threshold probability range from 0 to 1.0, while Rad-score and clinico-radiological model could add more net benefit with the range of 0–0.78 and 0.09–0.75, respectively (**Figure 7**). **Figure 8** showed the clinical use of the nomogram in two patients, who were both diagnosed with BI-RADS 4B category by radiologists.

## Discussion

The popularity of breast cancer screening has significantly increased the detection rate of small lesions, but making accurate qualitative diagnosis is still a challenge for radiologists. Our study discussed the potential ability of CESM-based radiomics in identifying benign and malignant breast lesions of sub-1 cm. Our results indicated that the radiomics nomogram combined with the radiomics signature and clinico-radiological variables could preoperatively predict the nature of small breast lesions with acceptable performance.

Radiomics is an emerging discipline based on traditional imaging examination and artificial intelligence. Radiomics features provide a stable and non-invasive approach to reflect the heterogeneity of lesions by revealing the texture features in depth. In this study, although the prediction accuracy of Rad-score was slightly lower than that of BI-RADS category, the radiomics nomogram combined with Rad-score was higher in accuracy and specificity than BI-RADS category only. This showed that radiomics could be used as an important supplement to clinico-radiological information in identifying benign and malignant small lesions. Luo et al. (22) extracted the radiomics features from the ultrasound images of BI-RADS category 4 and 5 patients, and analyzed the Rad-score containing 9 radiomics features and BI-RADS category, founding that the radiomics nomogram combined with Rad-score and BI-RADS category had the best predictive performance.

Gibbs et al. used radiomics analysis based on different MRI parameter maps to discriminate small benign and malignant breast lesions, yielding best AUC of 0.78 in the test set (23). A study by Lo et al. (24) conducted radiomics analysis on 96 BRCA-positive patients. They found that combining MRI-based radiomics with machine learning could improve the accuracy of the diagnosis of small breast masses in BRCA mutation carriers. And compared with the BI-RADS classification alone for assessment, radiomics could provide higher accuracy of 0.815. Our results also showed that CESM-based radiomics had good performance in identifying benign and malignant small breast lesions, and had AUCs of 0.903 and 0.868 in training and validation cohort, respectively.

The calibration curve is often used to verify the predictive effect of the prediction model by comparing actual probability with predictive probability. Our results showed that the predictive probability had high agreement with actual probability. In DCA, the theoretical relationship between the threshold probability and the relative values of false positive and false negative results was used to determine the clinical utility of the prediction model (25). In our study, DCA estimated the clinical value of radiomics nomogram and showed that the radiomics nomogram added more net benefit than “full treatment” or “none treatment”. This finding further confirmed that combining radiomics features with other available clinico-radiological data can improve the effectiveness of individual clinical decision making.

Our research has several advantages. First, to our knowledge, using CESM-based radiomics to identify benign and malignant breast lesions has not been previously reported. CESM has great advantages of showing calcification and reflecting blood flow, and can provide additional information on detecting breast diseases (26). Our results showed that CESM-based radiomics had better predictive performance in identifying benign and malignant breast lesions with an AUC of 0.940 compared with mammography-, ultrasound- and MRI-based radiomics (AUC=0.80, 0.928, 0,921) (22, 27, 28). Second, to ensure the reproducibility of feature extraction, only the features with ICCs>0.75 were qualified for the final analysis. Third, we used nomogram to predict benign and malignant small breast lesions. As a highly individualized visual prediction tool, nomogram has shown great potential in predicting disease progression and prognosis (29). The proposed nomogram could help clinicians choose the most appropriate treatment based on the predicted probability.

Admittedly, our study still has some limitations. First, the patients in this study were enrolled from a single institution with a limited number. Due to the small amount of patients in the validation cohort, this might have a certain impact on the validation of the proposed models. Mispredictions of a small number of lesions might cause significant difference. Despite the promising prospect of our results, a multicenter study with more balanced samples is warranted to prove the robustness of the proposed nomogram. Second, the ROIs were obtained manually; however, intra- and inter-correlation coefficients have shown good reproducibility in feature extraction. Previous studies have shown that the semi-automatic segmentation method could obtain relatively high intra- and inter-observer reproducibility (30, 31). Further work should use semi-automatic segmentation to draw the ROIs. Finally, the radiomics features were extracted on two-dimensional (2D) ROIs. Compared with three-dimensional (3D) features, 2D features may lose some important information that may fully describe the features of the entire lesion. However, studies have shown that 2D features had better performance than 3D features in lung cancer (32, 33).

In conclusion, the radiomics nomogram combined with CESM-based radiomics signature, BI-RADS category, and age demonstrated good predictive performance, calibration, and clinical utility in identifying benign and malignant breast lesions of sub-1 cm. CESM-based radiomics could serve as a potential tool to help clinicians make optimal clinical decision prior to biopsy or surgery and avoid overtreatment of benign lesions.

## Data Availability Statement

The raw data supporting the conclusions of this article will be made available by the authors, without undue reservation.

## Ethics Statements

The studies involving human participants were reviewed and approved by Yantai Yuhuangding Hospital, the affiliated hospital of Qingdao University. Written informed consent from the participants’ legal guardian/next of kin was not required to participate in this study in accordance with the national legislation and the institutional requirements.

## Author Contributions

FL, ZW, and NM: study design. FL, ML, QW, PY, and KZ: data collection. FL, YS, and JC: data processing. FL and ZW: manuscript writing. HX, HM: funding acquisition. All authors contributed to the article and approved the submitted version.

## Funding

This study was supported by the National Natural Science Foundation of China (82001775) and National Natural Science Foundation of China (81671654).

## Conflict of Interest

The authors declare that the research was conducted in the absence of any commercial or financial relationships that could be construed as a potential conflict of interest.
